# Receptor-Binding Domain Proteins of SARS-CoV-2 Variants Elicited Robust Antibody Responses Cross-Reacting with Wild-Type and Mutant Viruses in Mice

**DOI:** 10.3390/vaccines9121383

**Published:** 2021-11-24

**Authors:** Juan Shi, Xiaoxiao Jin, Yan Ding, Xiaotao Liu, Anran Shen, Yandan Wu, Min Peng, Chuanlai Shen

**Affiliations:** 1 Department of Microbiology and Immunology, Medical School of Southeast University, Nanjing 210009, China; 230198727@seu.edu.cn (J.S.); xiaoxiaojin@seu.edu.cn (X.J.); 230189322@seu.edu.cn (Y.D.); 220193625@seu.edu.cn (X.L.); 220203902@seu.edu.cn (Y.W.); pengminseu@163.com (M.P.); 2 Institute of Nephrology, Zhongda Hospital, Medical School of Southeast University, Nanjing 210009, China; 220193648@seu.edu.cn; 3 Jiangsu Province Key Laboratory of Critical Care Medicine, Department of Critical Care Medicine, Zhongda Hospital, Medical School of Southeast University, Nanjing 210009, China

**Keywords:** SARS-CoV-2, variants, spike protein, receptor-binding domain, immunogenicity, neutralizing antibodies

## Abstract

Multiple variants of severe acute respiratory syndrome coronavirus-2 (SARS-CoV-2) have spread around the world, but the neutralizing effects of antibodies induced by the existing vaccines have declined, which highlights the importance of developing vaccines against mutant virus strains. In this study, nine receptor-binding domain (RBD) proteins of the SARS-CoV-2 variants (B.1.1.7, B.1.351 and P.1 lineages) were constructed and fused with the Fc fragment of human IgG (RBD-Fc). These RBD-Fc proteins contained single or multiple amino acid substitutions at prevalent mutation points of spike protein, which enabled them to bind strongly to the polyclonal antibodies specific for wild-type RBD and to the recombinant human ACE2 protein. In the BALB/c, mice were immunized with the wild-type RBD-Fc protein first and boosted twice with the indicated mutant RBD-Fc proteins later. All mutant RBD-Fc proteins elicited high-level IgG antibodies and cross-neutralizing antibodies. The RBD-Fc proteins with multiple substitutions tended to induce higher antibody titers and neutralizing-antibody titers than the single-mutant RBD-Fc proteins. Meanwhile, both wild-type RBD-Fc protein and mutant RBD-Fc proteins induced significantly decreased neutralization capacity to the pseudovirus of B.1.351 and P.1 lineages than to the wild-type one. These data will facilitate the design and development of RBD-based subunit vaccines against SARS-COV-2 and its variants.

## 1. Introduction

Coronavirus Disease 2019 (COVID-19), which is caused by the severe acute respiratory syndrome coronavirus-2 (SARS-CoV-2), has rapidly disseminated across the world. As of 12 November 2021, the cumulative number of global cases exceeded 251 million, with 5.07 million deaths, making COVID-19 the most significant global health crisis since the 1918 influenza pandemic. Although existing progress in the popularization of vaccines has led to better control of the SARS-CoV-2 spread, many countries are experiencing a second or third wave of viral disease outbreaks as the virus mutates.

SARS-CoV-2 is prone to genetic evolution, thus leading to multiple variants. Some of these are classified by the World Health Organization (WHO) as variants of concern (VOCs), because they lead to increased transmissibility or virulence, increased difficulty of detection or reduced effectiveness of treatment or vaccines [1]. The current VOCs include B.1.1.7, B.1.351, P.1 and B.1.617.2 lineages, which harbor single or multiple mutations in the receptor-binding domain (RBD) of spike (S) glycoprotein [1,2,3,4,5,6]. The neutralization efficacy of antibodies induced by existing vaccines has declined when defending against these VOCs [7,8,9,10]. Developing new vaccines against SARS-CoV-2 variants is urgently needed.

Similar to other coronaviruses, the S protein of SARS-CoV-2 mediates virus entry into host cells and also serves as a target antigen inducing neutralizing antibodies production. It has a pre-fusion homotrimer structure, and each monomer consists of S1 and S2 subunits. After the RBD binds to the cellular receptor, and the angiotensin-converting enzyme 2 (ACE2) fixes to the host cells, the S1 subunit is naturally shed and the S2 subunit is exposed to form a stable post-fusion conformation [11,12,13]. COVID-19 vaccine candidates under development include inactivated virus vaccines, virus-like particle or nanoparticle vaccines, live-attenuated virus vaccines, protein-based subunit vaccines, virus-vectored vaccines, DNA vaccines and mRNA vaccines [14,15,16,17]. At present, a large number of SARS-CoV-2 vaccine candidates are subunit vaccines [14]. In this strategy, the RBD-based vaccine candidates have shown strong immunogenicity to elicit neutralizing antibodies production which could block the infections of the SARS-CoV-2 pseudovirus and live virus in vitro [18]. Moreover, the molecular size and immunogenicity of recombinant RBD proteins can be increased by fusing with an Fc fragment of human IgG [18,19]. Research focuses mainly on the mutant RBD proteins. The S protein with single mutation of V367F, N440K, S443A, E484R, S494P, N501Y, G502P and combinatorial mutations of S477N-E484K, E484K-N501Y or K417T-E484K-N501Y at RBD residues displayed a high binding affinity with the receptor ACE2, whereas the RBD proteins of A348T, V483A and K417N variants exhibited reduced affinity to ACE2 [20,21,22,23,24].

In this study, nine recombinant RBD proteins containing single or multiple new substitutions at the residues 417, 452, 484 and/or 501 of SARS-CoV-2 variants (B.1.1.7, B.1.351 and P.1 lineages) were constructed and fused with an Fc fragment of human IgG (RBD-Fc). The in vitro antigenicity, receptor-binding affinity and in vivo immunogenicity to elicit neutralizing antibodies were then investigated and compared with the wild-type RBD-Fc protein.

## 2. Materials and Methods

### 2.1. Mice and Ethical Approval

Female BALB/c mice aged between 6 and 8 weeks were purchased from the Comparative Medicine Center of Yangzhou University (Yangzhou, China) and maintained at the specific pathogen-free Animal Centre of Southeast University (Nanjing, China). After one week’s continuous assessment and acclimatization, the healthy mice (no weight loss, good appetite, normal behavior, smooth coat, black and grain-shaped stools) were selected for immunization experiments. The animal welfare and experimental procedures were approved by the Animal Ethics Committee of Southeast University (ref: 20190227001). All mouse-related experiments were performed in strict accordance with the Guidelines for the Care and Use of Laboratory Animals (Ministry of Science and Technology of China, 2006).

### 2.2. Preparation of Recombinant RBD Proteins

The wild-type RBD sequence of SARS-CoV-2 (residues 331 to 524) was amplified by PCR from a plasmid encoding the S protein of SARS-CoV-2 (GenBank accession number QHR63250.1) and fused with an Fc fragment of human IgG at the C terminal. The resulting recombinant plasmid (pSARS-CoV-2 RBD-Fc) was then used as the template to generate a series of plasmids containing single or multiple substitutions such as K417N, K417T, L452R, E484K, N501Y, K417N-E484K-N501Y, K417T-E484K-N501Y, K417N-L452R-E484K-N501Y or K417T-L452R-E484K-N501Y by using a QuikChange Multisite-Directed Mutagenesis kit (Agilent Technologies, Santa Clara, CA, USA). Each recombinant plasmid was routinely transfected into 293T cells (stored in-house) and followed by cell culture. The RBD-Fc proteins were purified from the culture supernatants by using nProtein A Sepharose 4 Fast Flow (GE Healthcare, Piscataway, NJ, USA).

### 2.3. SDS-PAGE and Western Blot

The purified proteins were separated by a 10% Tris-glycine SDS-PAGE, and then stained directly with Coomassie brilliant blue (for SDS-PAGE staining) or transferred to nitrocellulose membranes (BIO-RAD, Hercules, CA, USA) (for Western blot). The blots were blocked with 5% non-fat milk in PBST (where PBST contained PBS buffer with 0.05% of Tween 20) at 4 °C overnight, then further incubated with the sera from the mice immunized by wild-type RBD protein without an Fc fragment of human IgG (1:5000, prepared previously in-house) at 37 °C for 2 h, and followed by another 2 h incubation with horseradish peroxidase (HRP)-conjugated goat anti-mouse IgG (1:10,000, Abcam, Waltham, MA, USA). The signals were developed by ECL substrate reagents (BIO-RAD) and BIOMAX MR Film (Carestream, Rochester, NY, USA).

### 2.4. Mouse Immunization and Sample Collection

The wild-type RBD-Fc protein of SARS-CoV-2 (5 μg/mouse) was mixed with adjuvants aluminum (500 μg/mouse) and monophosphoryl lipid A (MPL, 10 μg/mouse) (InvivoGen, San Diego, CA, USA), and administered into BALB/c mice intramuscularly (i.m.), then the mice were i.m. boosted twice with wild-type RBD-Fc or the indicated mutant RBD-Fc protein (5 μg/mouse) together with aluminum (500 μg/mouse) and MPL (10 μg/mouse) at 3 weeks and 6 weeks after the first vaccination. Mice injected with the PBS plus adjuvants were used as a negative control. Facial vein blood or submandibular blood samples were collected for sera preparation 7 days after the final immunization. In total, 11 groups were designed with 10 mice for each group. Of these immunized mice, 5 mice from each group were used to collect sera for IgG antibody titers detection, while the other 5 mice were used to harvest sera for cross-neutralizing antibody titers detection.

### 2.5. ELISA

This assay was used to detect the IgG antibodies specific for the RBD protein of wild-type SARS-CoV-2 in the sera of immunized mice. Briefly, the ELISA plates were coated with the wild-type RBD protein without an Fc fragment of human IgG (1 μg/mL) at 4 °C overnight and blocked with 2% non-fat milk in PBST for 2 h at 37 °C. After washing with PBST three times, the plates were incubated with serially diluted mouse sera for 2 h at 37 °C and followed by incubation with HRP-conjugated goat anti-mouse IgG (1:5000), IgG1 (1:5000) or IgG2a (1:5000, Abcam) antibodies at 37 °C for 1 h. After further washing, the plates were then incubated with a 3,3′,5,5′-Tetramethylbenzidine (TMB) substrate (Sigma, Darmstadt, Germany), and the reaction was stopped using 1 N H_2_SO_4_. The absorbance at 450 nm (A450) was measured using an ELISA plate reader (Tecan, Männedorf, Switzerland). The A450 value in the experimental well containing sera (A450_Exp_) was subtracted at the A450 value in the negative control well without sera (A450_Neg_). Then, the calibrated A450_Exp_ value and sera dilution times in each experimental well were used to generate the standard curve using GraphPad Prism 9. The A450_Exp_/A450_Neg_ ≥ 4 was defined as the cut-off line of positive binding between the antibody and antigen. The highest dilution time of sera while the A450_Exp_/A450_Neg_ ≥ 4 was calculated and determined was the RBD protein-specific antibody titer in the immunized mouse.

Similarly, the binding of RBD-Fc proteins with human ACE2 (hACE2) protein was also detected by using an ELISA protocol. Briefly, the ELISA plates were coated with wild-type RBD-Fc protein or each kind of mutant RBD-Fc proteins (2 μg/mL) first and incubated with hACE2 protein (20 or 5 μg/mL, Abcam) later. Then, the reactions were detected using goat anti-hACE2 IgG (0.2 μg/mL, R&D system, Minneapolis, MN, USA) and HRP-conjugated chicken anti-goat IgG antibody (1:5000, Abcam).

### 2.6. Generation of Wild-Type and Mutant SARS-CoV-2 Pseudoviruses and Neutralization Experiment

SARS-CoV-2 pseudoviruses were generated by co-transfecting 293T cells with the lentiviral vector plasmids named pLenti-CMV-Luciferase and PS-PAX2 (stored in our laboratory), as well as the plasmid encoding the wild-type S protein or mutant S proteins (K417N-E484K-N501Y or K417T-E484K-N501Y) of SARS-CoV-2 (generated in-house), using the calcium phosphate method. The culture medium was replaced with fresh Dulbecco’s Modified Eagle Medium (DMEM) 6 h later. The supernatants containing the indicated pseudovirus were collected 72 h after transfection, and used for single-cycle infection. For pseudovirus neutralization experiments, the SARS-CoV-2 pseudoviruses expressing wild-type or each mutant S protein were incubated for 2 h at 37 °C with the heat-inactivated and serially diluted sera of immunized mice and then co-cultured with the 293T-hACE2 cells (from BEI Resources as item NR-52511; https://www.beiresources.org/, accessed on 28 July 2021) that express human ACE2; validated as a consistent and effective cell line for SARS-CoV-2 pseudovirus neutralization assays [25,26]. After 72 h co-cultures, the cell lystes were prepared using cell lysis buffer (Promega, Madison, USA) and incubated with luciferase substrate (Promega). The relative luciferase activity was measured by using the Infinite 200 PRO Luminometer (Tecan). Viral infectivity was assessed by comparing the relative luciferase activities between the virus well alone and the wells of the virus along with the sera of the immunized mice. The inhibitory activity of sera from the vaccinated mouse to the blocking pseudovirus infection was determined by comparing the average luciferase activity between the virus well with the sera of RBD-Fc vaccinated mice and the virus well with the sera of PBS-injected mice. Then, the neutralizing antibody titer in each immunized mouse was defined as the highest sera dilution times by which the viral infectivity was neutralized by 50% (NT_50_). NT_50_ values were calculated using non-linear regression in GraphPad Prism.

### 2.7. Statistical Analysis

Statistical significance was calculated across groups by Two-way ANOVA and multiple comparisons using GraphPad Prism 9 (GraphPad, La Jolla, CA, USA). *p* < 0.05 was considered as significant. Asterisks (* and **) in the figures denote *p* values less than 0.05 and 0.01, respectively. 

## 3. Results

### 3.1. Characterization of Recombinant Mutant RBD-Fc Proteins of SARS-CoV-2 

**Figure 1:**
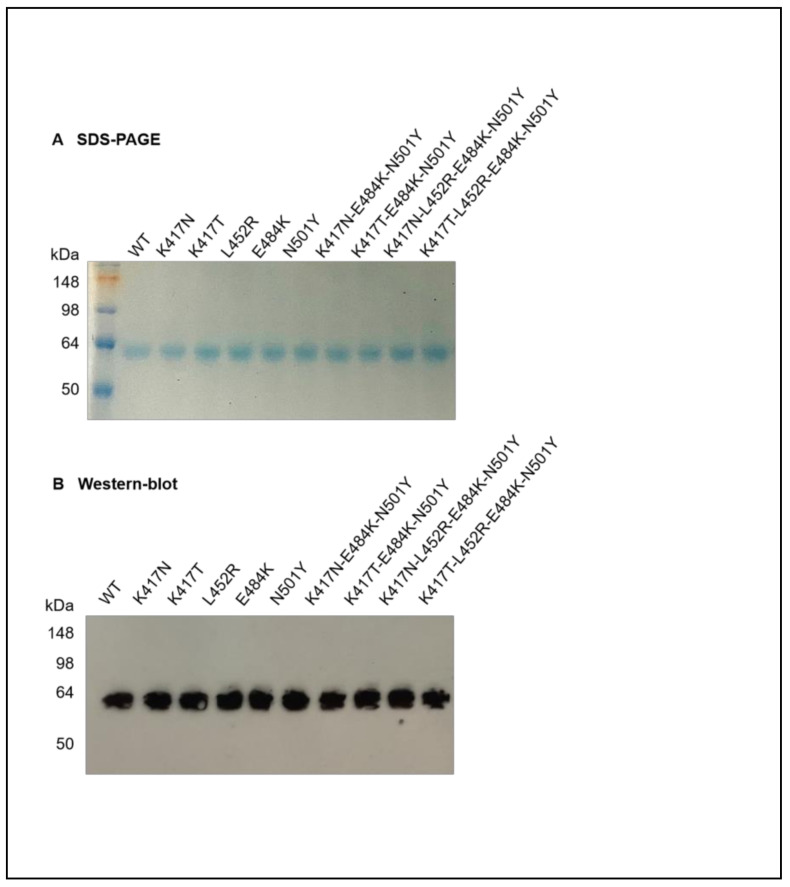
Characterization of recombinant mutant RBD-Fc proteins of SARS-CoV-2. Nine mutant RBD-Fc proteins and wild-type RBD-Fc protein were expressed in 239T cells and purified from the cell culture supernatants. Each protein was then subjected to SDS-PAGE for Coomassie brilliant blue staining (**A**) and for Western blot (**B**) with the sera of mice immunized by wild-type RBD protein without the Fc fragment of human IgG. WT: wild-type RBD-Fc protein.

Nine recombinant RBD-Fc plasmids containing single or multiple mutations of K417N, K417T, L452R, E484K, N501Y, K417N-E484K-N501Y, K417T-E484K-N501Y, K417N-L452R-E484K-N501Y or K417T-L452R-E484K-N501Y in the RBD of SARS-CoV-2 variants (B.1.1.7, B.1.351 and P.1 lineages) were structured, identified by gene sequencing and expressed in 293T cells. The SDS-PAGE showed that all the mutant RBD-Fc proteins collected from the culture supernatants of 293T cells possessed a molecule size similar to the 57kDa of wild-type RBD-Fc protein, and with high purity (Figure 1A). The Western blot assay indicated that all the mutant RBD-Fc proteins reacted strongly with the RBD-specific polyclonal antibodies in the sera of mice vaccinated by wild-type RBD protein without an Fc fragment of human IgG (Figure 1B). The uncropped blots were shown in Figure S1. These results suggested the good antigenicity of mutant RBD-Fc proteins similar to wild-type RBD-Fc protein in vitro.

### 3.2. Binding Affinity of Mutant SARS-CoV-2 RBD-Fc Proteins with hACE2

**Figure 2:**
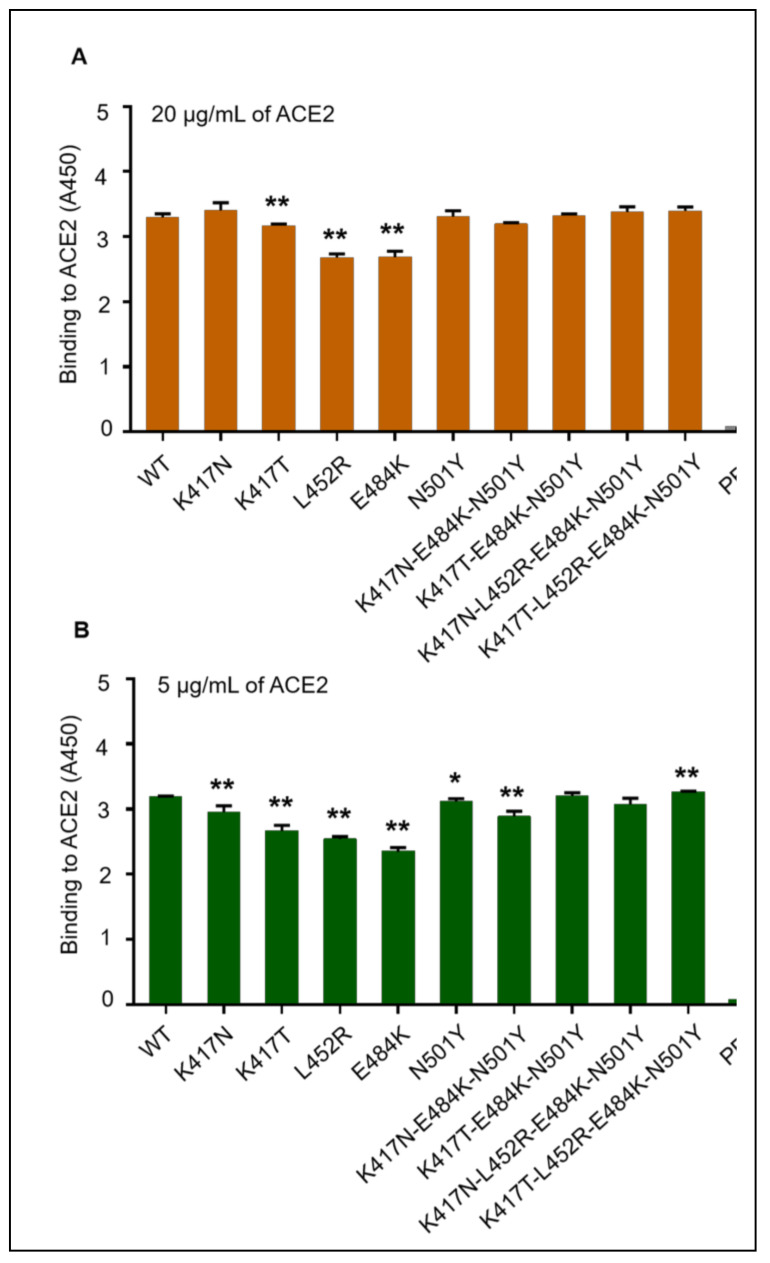
Binding affinity of mutant SARS-CoV-2 RBD-Fc proteins with hACE2. Wild-type RBD-Fc protein and each mutant RBD-Fc protein were coated in 96-well plate respectively, and then reacted with recombinant hACE2 protein in ELISA. (**A**) Binding of each RBD-Fc protein with 20 μg/mL of hACE2 protein; (**B**) binding of each RBD-Fc Protein with 5 μg/mL of hACE2 protein. The data of A450 in each group were presented as mean ± standard deviation (SD) of four replicate wells. * (*p* < 0.05) and ** (*p* < 0.01) mean significant differences between the mutant RBD-Fc protein and wild-type RBD-Fc protein. WT: wild-type RBD-Fc protein; PBS: PBS solution without RBD protein.

To evaluate the binding affinity of recombinant mutant RBD-Fc proteins with hACE2, the cellular binding receptor onto host cells of SARS-CoV-2, an ELISA test was performed. Each mutant RBD-Fc protein displayed a strong binding affinity to the soluble hACE2 protein at the concentrations of 20 μg/mL (Figure 2A) or 5 μg/mL (Figure 2B), but K417N, K417T, L452R, E484K and K417N-E484K-N501Y mutant proteins showed a significantly lower affinity with hACE2 than the wild-type RBD-Fc protein (Figure 2B). These data suggested that, although the nine mutant RBD-Fc proteins maintained good functionality in binding to the SARS-CoV-2 receptor, the substitutions of K417N, K417T, L452R or E484K of RBD may decrease the affinity between ligand and receptor in vivo.

### 3.3. Mutant RBD-Fc Proteins of SARS-CoV-2 Elicited Robust Antibody Responses in Mice

**Figure 3:**
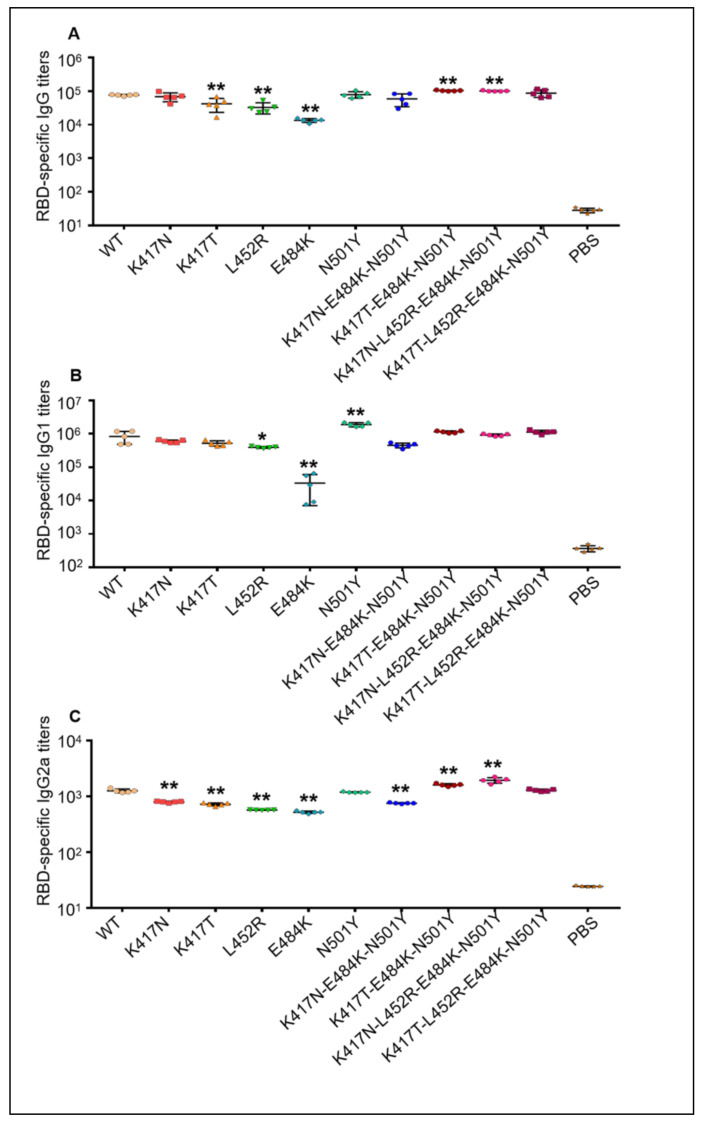
Mutant RBD-Fc proteins of SARS-CoV-2 elicited robust antibody responses in mice. BALB/c mice were immunized with wild-type RBD-Fc protein first and then boosted twice with the indicated mutant RBD-Fc proteins later. Seven days after the final vaccination, the mice sera were collected and subjected to a series of dilutions, followed by the detection of antibodies binding to the wild-type RBD protein without the Fc fragment of human IgG in ELISA. The A450 value in the experimental well containing sera (A450_Exp_) was subtracted at the A450 value in the negative control well without sera (A450_Neg_). Then, the calibrated A450_Exp_ value and dilution times of the sera in each experimental well were used to generate the standard curve using GraphPad Prism 9. The highest dilution time of sera while the A450_Exp_/A450_Neg_ ≥ 4 was calculated and determined was the RBD protein-specific antibody titer in the immunized mice. (**A**) RBD-specific IgG antibody titers in each immunized mouse; (**B**) RBD-specific IgG1 antibody titers in each immunized mouse; (**C**) RBD-specific IgG2a antibody titers in each immunized mouse. The antibody titers were expressed as mean ± SD of five mice in each group. * (*p* < 0.05) and ** (*p* < 0.01) mean significant differences between the mutant RBD-Fc protein booster group and wild-type RBD-Fc protein vaccination group. WT: the mice group immunized three times with wild-type RBD-Fc protein; PBS: negative control group injected with PBS plus adjuvants.

To investigate the in vivo immunogenicity of mutant RBD-Fc proteins, BALB/c mice were immunized with wild-type RBD-Fc protein first and boosted twice with the indicated mutant RBD-Fc proteins later. Then, the mice sera were collected 7 days after the final vaccination and followed by the detection of RBD-specific IgG, IgG1 and IgG2a antibodies by ELISA. As shown in Figure 3A, all groups immunized by wild-type RBD-Fc protein or boosted by mutant RBD-Fc proteins displayed high-titer IgG antibodies binding to the wild-type RBD protein without an Fc fragment of human IgG. As compared with the mice immunized three times by wild-type RBD-Fc protein, the mice boosted twice with K417T, L452R or E484K mutant RBD-Fc proteins showed significantly lower IgG antibody titers (*p* < 0.01), while the mice boosted twice with K417T-E484K-N501Y or K417N-L452R-E484K-N501Y mutant RBD-Fc proteins exhibited considerably higher IgG antibody titers (*p* < 0.01). In parallel, only a background level of IgG antibodies bound to the wild-type RBD protein in the controlled mice was injected with PBS plus adjuvants. Furthermore, the levels of IgG1 antibodies binding to the wild-type RBD protein without an Fc fragment (Figure 3B) were much higher than IgG2a levels (Figure 3C) in each vaccination group, implying the Th2 type immune response was elicited by the RBD-Fc proteins. Similarly, partial RBD-Fc proteins containing single mutant reside elicited lower IgG1 or IgG2 antibodies titers, while partial ones containing multiple mutant resides elicited higher IgG1 or IgG2a antibodies titers, when compared to the wild-type RBD-Fc protein immunizations. These data demonstrated the strong immunogenicity of these mutant RBD-Fc proteins by inducing robust humoral immune responses in mice. 

In the process of the experiment, the mice were monitored twice a week for feces and urine, reluctance to move, anorexia, dehydration, 10% weight loss, fur ruffling, skin condition, lesions, abscess and normal behavior patterns. No adverse reactions were found in the immunized mice and there was no mortality during the process. However, no mouse organ was collected for the hematoxylin–eosin staining in this study.

### 3.4. Mutant RBD-Fc Proteins of SARS-CoV-2 Induced High-Level Cross-Neutralizing Antibodies Production in Mice

To verify whether mutant RBD-Fc proteins induced highly effective neutralizing antibody production in vivo, the pseudovirus neutralization experiments were carried out. The sera from each immunized mouse were co-incubated with the SARS-CoV-2 pseudoviruses which express the S protein of wild-type virus or variant strains (K417N-E484K-N501Y or K417T-E484K-N501Y) followed by co-cultures with the 293T-hACE2 cells. 72 h later, the cell lyste was prepared and the pseudovirus was detected by measuring the relative luciferase activity. The neutralizing antibody titers needed to neutralize 50% of viral infectivity (NT_50_) were calculated using non-linear regression in GraphPad Prism.

**Figure 4:**
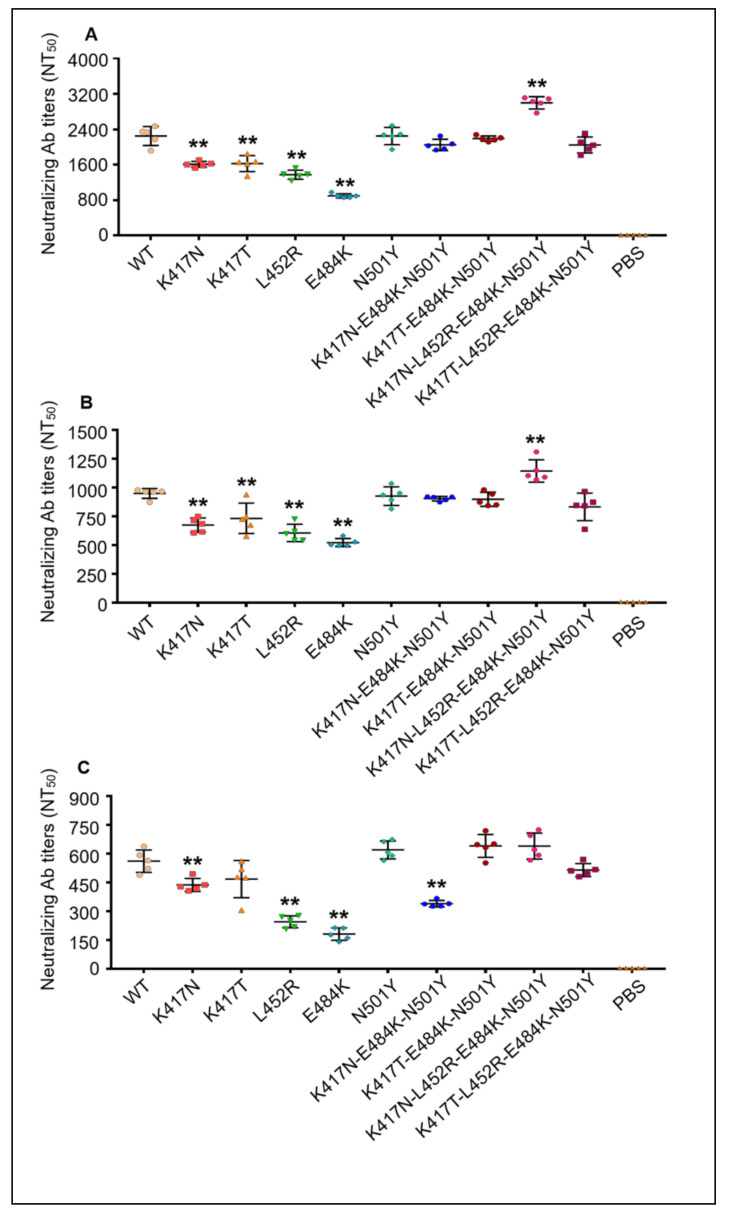
Mutant RBD-Fc proteins of SARS-CoV-2 induced the production of high-level cross-neutralizing antibodies in mice. Mice sera were collected from each immunized BALB/c mice at 7 days after final vaccination, subjected to a series of dilutions, and used in the neutralization experiments of SARS-CoV-2 pseudoviruses. The neutralizing antibody titer in each immunized group was defined as the sera dilution times by which the viral infectivity was neutralized by 50% (NT_50_). Each dot represented the NT_50_ value of each individual mouse, and the data were presented as mean ± SD for five mice in each group. (**A**) Neutralizing antibody titers against the SARS-CoV-2 pseudoviruses expressing wild-type S protein; (**B**) neutralizing antibody titers against the SARS-CoV-2 pseudoviruses expressing mutant S protein of K417N-E484K-N501Y (B.1.351 lineage); (**C**) neutralizing antibody titers against the SARS-CoV-2 pseudoviruses expressing mutant S protein of K417T-E484K-N501Y (P.1 lineage). ** (*p* < 0.01) means significant differences between the mutant RBD-Fc protein booster group and wild-type RBD-Fc protein vaccination group. WT: the mice group immunized three times with wild-type RBD-Fc protein; PBS: negative control group injected with PBS plus adjuvants.

As shown in Figure 4, both wild-type and the mutant RBD-Fc proteins induced obvious production of neutralizing antibodies against SARS2-CoV-2 pseudovirus, which express the wild-type S protein (Figure 4A), the S protein with mutations of K417N-E484K-N501Y (B.1.351 lineage) (Figure 4B) or the S protein with mutants of K417T-E484K-N501Y (P.1 lineage) (Figure 4C), with the levels of neutralizing antibodies (NT_50_) of 1929.95 ± 569.20, 817.51 ± 191.99 and 463.92 ± 164.27, respectively. Noticeably, the neutralizing effects were much stronger in the wild-type SARS2-CoV-2 pseudovirus than in the pseudoviruses of the two variants. Meanwhile, as compared with the wild-type RBD-Fc protein, the mutant RBD-Fc proteins containing single amino acid substitution (K417N, K417T, L452R and E484K) elicited significantly lower-level neutralizing antibodies, while the RBD-Fc protein with multiple amino acid mutations (K417N-L452R-E484K-N501Y) elicited higher-level neutralizing antibodies, which defended against the wild-type pseudovirus and mutant pseudovirus. These data indicate that the new mutant RBD-Fc proteins of SARS-CoV-2 could induce the production of antibodies that cross-neutralize the wild-type virus and the two variants in the mice model, but both the wild-type RBD-Fc protein and the mutant RBD-Fc proteins elicited lower-level neutralizing antibodies against the two variants than against the wild-type virus.

## 4. Discussion

Developing safe and highly effective vaccines to prevent persistent human infection of SARS-CoV-2 and its variants has become the most important priority. Recombinant protein-based subunit vaccines are considered to have good safety [14]. An RBD fragment containing residues 331–524 of S protein has been demonstrated as a critical neutralizing domain capable of inducing high-level neutralizing antibodies against the SARS-CoV-2 infection and thus serves as an important target for the development of subunit vaccines [14,18,19]. Particularly, an RBD vaccine, a shorter fragment than the full-length S protein, is expected to effectively induce neutralizing antibody production and minimize the potential for producing non-neutralizing antibodies. 

Unlike previous research on RBD mutations [20,21,22,23,24], this study investigated the nine RBD-Fc proteins with new mutants (K417N, K417T, L452R, E484K, N501Y, K417N-E484K-N501Y, K417T-E484K-N501Y, K417N-L452R-E484K-N501Y or K417T-L452R-E484K-N501Y) from the SARS-CoV-2 variants of B.1.1.7, B.1.351 and P.1 lineages, and compared them with the RBD-Fc protein containing the sequence of prototypic SARS-CoV-2 strain. All the mutant RBD-Fc proteins demonstrated strong reactions with the wild-type RBD-specific polyclonal antibodies and a high binding affinity with the hACE2 protein. As compared with the wild-type RBD-Fc protein, the mutant RBD-Fc with mutations of K417N, K417T, L452R and E484K showed a reduced binding affinity with the hACE2 protein, which is partially consistent with the previous reports such as the A348T, V483A and K417N variants [21,24].

To date, many studies have confirmed that the neutralizing antibodies induced by existing vaccines, or vaccine candidates, and the neutralizing antibodies in the early convalescent patients with COVID-19, displayed reduced cross-neutralizing effects on the SARS-CoV-2 variants to different extents [7,8,9,10,27,28,29,30]. For example, the human sera induced by the BNT162b2 and mRNA-1273 vaccines displayed cross-neutralizing to a B.1.351 lineage reduced by 6.5–8.6-fold for the pseudovirus and 10.3–12.4-fold for the live virus [8]. Human sera elicited by the BNT162b2 vaccine has shown a neutralizing capacity to the B.1.1.7 lineage similar to the wild-type strain, but a significantly lower neutralization capacity to the P.1 variant (mean NT_50_ value 3.3-fold lower) [9]. In addition, a lentiviral pseudovirus test, which was conducted by using the 293T-hACE2 cells and a relatively homogeneous group of hospitalized convalescent sera from severe COVID-19, has reported an average 4.8-fold reduction in cross-neutralization to the B.1.351 lineage [29]. The convalescent sera from patients infected with the wild-type virus have poor neutralizing effects on the B.1.1.7 and P.1 lineages when compared to the 20A.EU1 strain (mean NT_50_ value 1.6- and 6.7-fold lower, respectively) [9]. These reports suggest the importance of developing effective vaccines against the SARS-CoV-2 variants. 

In this study, all the mutant RBD-Fc proteins maintained a strong in vivo immunogenicity in eliciting robust humoral immune responses and cross-neutralizing antibodies that inhibited the infections of the SARS-CoV-2 pseudovirus expressing wild-type or mutant S proteins. However, consistent with the previous studies [9,29], both the wild-type RBD-Fc protein immunization and the mutant RBD-Fc proteins boosters induced lower-level neutralizing antibodies against the pseudovirus of B.1.351 and P.1 lineages than against the wild-type one (mean NT_50_ value 2.36 and 4.16-fold lower, respectively). Whether the three-time immunizations of mutant RBD-Fc proteins can elicit comparable levels for neutralizing antibodies against the mutant virus and the wild-type virus remains to be further investigated. To determine whether the boosters with the mutant RBD vaccines—after the vaccination with the existing wild-type vaccine—can elicit the production of antibodies specific for the SARS-CoV-2 variants, mice were immunized with the wild-type RBD-Fc protein first and boosted twice with the indicated mutant RBD-Fc protein later. This vaccination regimen, to some extent, can simulate the booster immunization using mutant RBD vaccines for the population previously injected with the vaccines against the prototypic SARS-CoV-2 strain.

Of note is that the mutant RBD-Fc proteins containing a single substitution (K417N, K417T, L452R and E484K) displayed reduced immunogenicity while the protein with multiple substitutions (K417N-L452R-E484K-N501Y) showed increased immunogenicity in eliciting cross-neutralizing antibody production, as compared with the wild-type RBD-Fc protein. Further molecule docking and dynamic simulation assays are needed for the design of RBD-based vaccines against SARS-CoV-2 variants.

In addition, two limitations in this study must be mentioned. The specific cellular immunity was not evaluated after the RBD-Fc proteins vaccination. For the protective effects of the vaccine candidates, more reliable results can be obtained from the neutralization experiment using the live virus in vitro and the virus challenge experiments in immunized mice. In our future work, more mutant RBD proteins will be designed according to the current SARS-CoV-2 variants. Glycosylation modification for all the mutant RBD proteins will be performed to enhance their immunogenicity in eliciting cross-neutralizing antibodies and specific T cell responses. After that, virus challenge experiments in mice will be carried out to identify the in vivo protective efficacy of these modified mutant RBD-Fc proteins.

## 5. Conclusions

Here, nine RBD proteins of SARS-CoV-2 variants were constructed and fused with the Fc fragment of human IgG. These RBD-Fc proteins contained single or multiple amino acid substitutions that were recognized by the polyclonal antibodies specific for the wild-type RBD and could bind strongly to the recombinant hACE2 protein. In mice immunized with the wild-type RBD-Fc protein first and boosted twice with the mutant RBD-Fc protein later, all the mutant RBD-Fc proteins elicited high-level IgG antibodies and cross-neutralizing antibodies. The RBD-Fc proteins with multiple substitutions tended to induce higher antibody titers—and neutralizing antibody titers—than the single-mutant RBD-Fc proteins. Meanwhile, both wild-type RBD-Fc protein and mutant RBD-Fc proteins produced a significantly decreased neutralization capacity to the pseudovirus of B.1.351 and P.1 lineages than against6 the pseudovirus expressing the wild-type S protein. These findings will aid in the development of RBD-based subunit vaccines against SARS-CoV-2 and its variants.

## Data Availability

Data are contained within the article.

## Supplementary Materials

The following are available online at https://www.mdpi.com/article/10.3390/vaccines9121383/s1, Figure S1: The binding of recombinant mutant RBD-Fc proteins of SARS-CoV-2 with the sera of mice immunized by wild-type RBD protein in Western blot (uncropped blots).

## Abbreviations

COVID-19	Coronavirus Disease 2019
SARS-CoV-2	Severe acute respiratory syndrome coronavirus-2
VOCs	Variants of concern
RBD	Receptor-binding domain
hACE2	Human angiotensin-converting enzyme 2
HRP	Horseradish peroxidase
MPL	Monophosphoryl lipid A
DMEM	Dulbecco’s Modified Eagle Medium
NT50	Neutralizing antibody titer needed to neutralize 50% of viral infectivity.

## References

[1] World Health Organization (WHO) Tracking SARS-CoV-2 Variants. https://www.who.int/en/activities/tracking-SARS-CoV-2-variants/.

[2] Cascella M., Rajnik M., Aleem A., Dulebohn S.C., Di Napoli R. (2021). Features, Evaluation, and Treatment of Coronavirus (COVID-19).

[3] Galloway S.E., Paul P., MacCannell D.R., Johansson M.A., Brooks J.T., MacNeil A., Slayton R.B., Tong S., Silk B.J., Armstrong G.L. (2021). Emergence of SARS-CoV-2 B.1.1.7 Lineage—United States, December 29, 2020–January 12, 2021. MMWR. Morb. Mortal. Wkly. Rep..

[4] Volz E., Mishra S., Chand M., Barrett J.C., Johnson R., Geidelberg L., Hinsley W.R., Laydon D.J., Dabrera G., O’Toole Á. (2021). Assessing transmissibility of SARS-CoV-2 lineage B.1.1.7 in England. Nature.

[5] Tegally H., Wilkinson E., Giovanetti M., Iranzadeh A., Fonseca V., Giandhari J., Doolabh D., Pillay S., San E.J., Msomi N. (2021). Detection of a SARS-CoV-2 variant of concern in South Africa. Nature.

[6] Faria N.R., Mellan T.A., Whittaker C., Claro I.M., Candido D.D.S., Mishra S., Crispim M.A.E., Sales F.C., Hawryluk I., McCrone J.T. (2021). Genomics and Epidemiology of a Novel SARS-CoV-2 Lineage in Manaus, Brazil. MedRxiv.

[7] Cele S., Africa N.F.G.S.I.S., Gazy I., Jackson L., Hwa S.-H., Tegally H., Lustig G., Giandhari J., Pillay S., Wilkinson E. (2021). Escape of SARS-CoV-2 501Y.V2 from neutralization by convalescent plasma. Nature.

[8] Wang P., Casner R.G., Nair M.S., Wang M., Yu J., Cerutti G., Liu L., Kwong P.D., Huang Y., Shapiro L. (2021). Increased resistance of SARS-CoV-2 variant P.1 to antibody neutralization. Cell Host Microbe.

[9] Gidari A., Sabbatini S., Bastianelli S., Pierucci S., Busti C., Monari C., Pasqua B.L., Dragoni F., Schiaroli E., Zazzi M. (2021). Cross-neutralization of SARS-CoV-2 B.1.1.7 and P.1 variants in vaccinated, convalescent and P.1 infected. J. Infect..

[10] Liu C., Ginn H.M., Dejnirattisai W., Supasa P., Wang B., Tuekprakhon A., Nutalai R., Zhou D., Mentzer A.J., Zhao Y. (2021). Reduced neutralization of SARS-CoV-2 B.1.617 by vaccine and convalescent serum. Cell.

[11] Benton D.J., Wrobel A.G., Xu P., Roustan C., Martin S.R., Rosenthal P.B., Skehel J.J., Gamblin S.J. (2020). Receptor binding and priming of the spike protein of SARS-CoV-2 for membrane fusion. Nature.

[12] Nguyen H.T., Zhang S., Wang Q., Anang S., Wang J., Ding H., Kappes J.C., Sodroski J. (2021). Spike Glycoprotein and Host Cell Determinants of SARS-CoV-2 Entry and Cytopathic Effects. J. Virol..

[13] Walls A.C., Park Y.-J., Tortorici M.A., Wall A., McGuire A.T., Veesler D. (2020). Structure, Function, and Antigenicity of the SARS-CoV-2 Spike Glycoprotein. Cell.

[14] Dai L., Gao G.F. (2021). Viral targets for vaccines against COVID-19. Nat. Rev. Immunol..

[15] Ura T., Yamashita A., Mizuki N., Okuda K., Shimada M. (2021). New vaccine production platforms used in developing SARS-CoV-2 vaccine candidates. Vaccine.

[16] Li L., Guo P., Zhang X., Yu Z., Zhang W., Sun H. (2021). SARS-CoV-2 vaccine candidates in rapid development. Hum. Vaccines Immunother..

[17] Russell R.L., Pelka P., Mark B.L. (2021). Frontrunners in the race to develop a SARS-CoV-2 vaccine. Can. J. Microbiol..

[18] Yang J., Wang W., Chen Z., Lu S., Yang F., Bi Z., Bao L., Mo F., Li X., Huang Y. (2020). A vaccine targeting the RBD of the S protein of SARS-CoV-2 induces protective immunity. Nature.

[19] Liu Z., Xu W., Xia S., Gu C., Wang X., Wang Q., Zhou J., Wu Y., Cai X., Qu D. (2020). RBD-Fc-based COVID-19 vaccine candidate induces highly potent SARS-CoV-2 neutralizing antibody response. Signal. Transduct. Target. Ther..

[20] Gu H., Chen Q., Yang G., He L., Fan H., Deng Y.-Q., Wang Y., Teng Y., Zhao Z., Cui Y. (2020). Adaptation of SARS-CoV-2 in BALB/c mice for testing vaccine efficacy. Science.

[21] Chakraborty S. (2021). Evolutionary and structural analysis elucidates mutations on SARS-CoV2 spike protein with altered human ACE2 binding affinity. Biochem. Biophys. Res. Commun..

[22] Han Y., Wang Z., Wei Z., Schapiro I., Li J. (2021). Binding affinity and mechanisms of SARS-CoV-2 variants. Comput. Struct. Biotechnol. J..

[23] Boehm E., Kronig I., Neher R.A., Eckerle I., Vetter P., Kaiser L. (2021). Novel SARS-CoV-2 variants: The pandemics within the pandemic. Clin. Microbiol. Infect..

[24] Laffeber C., de Koning K., Kanaar R., Lebbink J.H. (2021). Experimental Evidence for Enhanced Receptor Binding by Rapidly Spreading SARS-CoV-2 Variants. J. Mol. Biol..

[25] Schmidt F., Weisblum Y., Muecksch F., Hoffmann H.-H., Michailidis E., Lorenzi J.C., Mendoza P., Rutkowska M., Bednarski E., Gaebler C. (2020). Measuring SARS-CoV-2 neutralizing antibody activity using pseudotyped and chimeric viruses. J. Exp. Med..

[26] Crawford K.H.D., Eguia R., Dingens A.S., Loes A.N., Malone K.D., Wolf C.R., Chu H.Y., Tortorici M.A., Veesler D., Murphy M. (2020). Protocol and Reagents for Pseudotyping Lentiviral Particles with SARS-CoV-2 Spike Protein for Neutralization Assays. Viruses.

[27] Amanat F., Strohmeier S., Lee W.-H., Bangaru S., Ward A.B., Coughlan L., Krammer F. (2021). Murine Monoclonal Antibodies against the Receptor Binding Domain of SARS-CoV-2 Neutralize Authentic Wild-Type SARS-CoV-2 as Well as B.1.1.7 and B.1.351 Viruses and Protect In Vivo in a Mouse Model in a Neutralization-Dependent Manner. MBio.

[28] Tada T., Dcosta B.M., Zhou H., Vaill A., Kazmierski W., Landau N.R. (2021). Decreased neutralization of SARS-CoV-2 global variants by therapeutic anti-spike protein monoclonal antibodies. BioRxiv.

[29] Vidal S.J., Collier A.-R.Y., Yu J., McMahan K., Tostanoski L.H., Ventura J.D., Aid M., Peter L., Jacob-Dolan C., Anioke T. (2021). Correlates of Neutralization against SARS-CoV-2 Variants of Concern by Early Pandemic Sera. J. Virol..

[30] Liu Z., VanBlargan L.A., Bloyet L.M., Rothlauf P.W., Chen R.E., Stumpf S., Zhao H., Errico J.M., Theel E.S., Liebeskind M.J. (2021). Identification of SARS-CoV-2 spike mutations that attenuate monoclonal and serum antibody neutralization. Cell Host Microbe.

