# Supplementary material for: Receptor-Binding Domain Proteins of SARS-CoV-2 Variants Elicited Robust Antibody Responses Cross-Reacting with Wild-Type and Mutant Viruses in Mice
Source: Vaccines (Basel). 2021 Nov 24;9(12):1383. doi: 10.3390/vaccines9121383 (PMC8705015; doi:10.3390/vaccines9121383)
Supplement: Supplementary file 1 [file vaccines-09-01383-s001.zip › vaccines-1445051-supplementary.pdf]

Supplementary Information

The purified recombinant RBD-Fc proteins were separated by a 10% Tris-glycine SDS-PAGE, and then transferred to nitrocellulose membranes (BIO-RAD, Hercules, CA, USA) (for Western blot). The blots were blocked with 5% non-fat milk in PBST (where PBST contained PBS buffer with 0.05% of Tween 20) at 4 °C overnight, then further incubated with the sera from the mice immunized by wild-type RBD protein without an Fc fragment of human IgG (1:5000, prepared previously in-house) at 37 °C for 2 h, and followed by another 2 h incubation with horseradish peroxidase (HRP)-conjugated goat anti-mouse IgG (1:10,000, Abcam, Waltham, MA, USA). The signals were developed by ECL substrate reagents (BIO-RAD) and BIOMAX MR Film (Carestream, Rochester, NY, USA) in the dark room. As protein marker could not be developed, the position of marker was manually marked after comparing the developed film and nitrocellulose membrane.

Supplementary Figure S1:

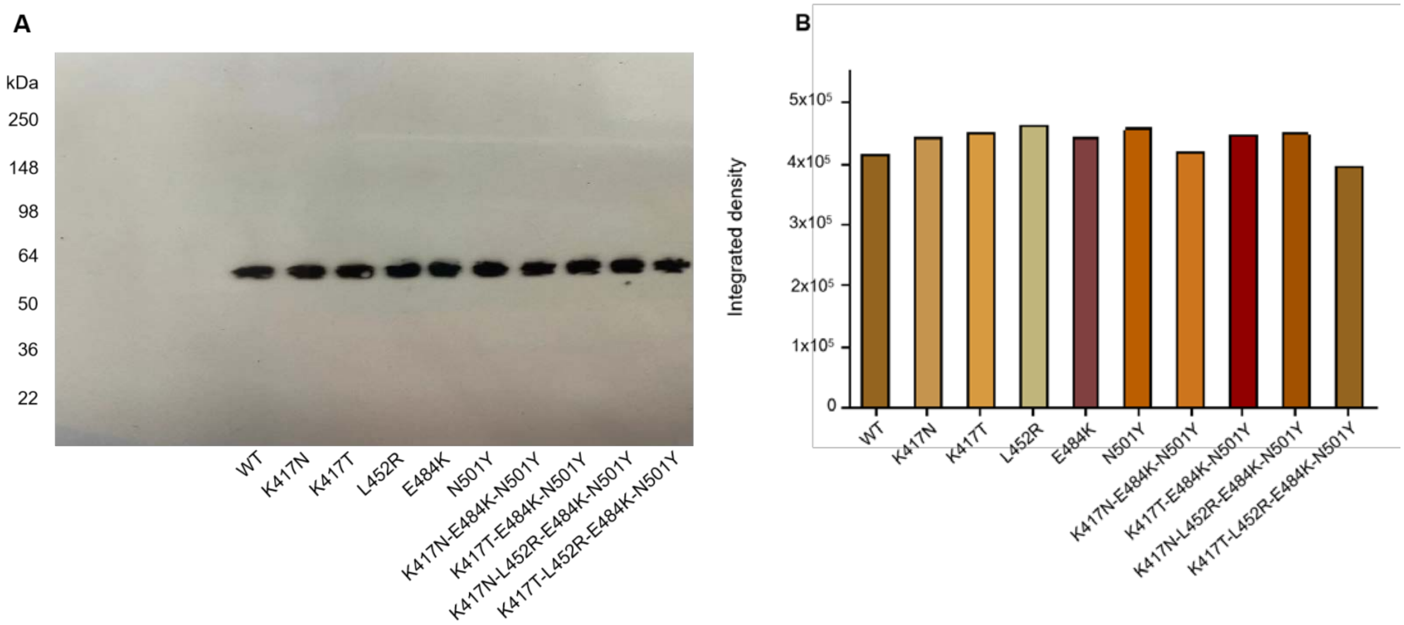

Figure S1: The binding of recombinant mutant RBD-Fc proteins of SARS-CoV-2 with the sera of mice immunized by wild-type RBD protein in Western blot (uncropped blots). (**A**) Western blots. Nine mutant RBD-Fc proteins and wild-type RBD-Fc protein were expressed in 239T cells and purified from the cell culture supernatants. Each protein was then subjected to SDS-PAGE for Western blot with the sera of mice immunized by wild-type RBD protein without the Fc fragment of human IgG. (**B**) Density of blot bands. The integrated density of each band was analyzed by ImageJ. The integrated density was simply the area and mean gray value multiplied together for each band.
